# Effects of blood flow restriction combined with high-load training on muscle strength and sports performance in athletes: a systematic review and meta-analysis

**DOI:** 10.3389/fphys.2025.1603568

**Published:** 2025-07-02

**Authors:** Chentianlei Su, Zhenglong Zhang, Bin Liang, Sicen Zhou, Xingyu Long

**Affiliations:** ^1^ School of Physical Education, Chongqing Technology and Business University, Chongqing, China; ^2^ Graduate School, Harbin Sport University, Harbin, Heilongjiang, China

**Keywords:** blood flow restricted training, athlete, meta-analysis, high-load training, systematic review

## Abstract

**Objective:**

This study aims to present updated convergent analyses and data following systematic review and meta-analysis protocols to determine the effects of high-load resistance training (HL-RT) combined with blood flow restriction (BFR) on athletes’ physiological adaptations (muscle strength and body composition) and athletic performance (power, speed, and endurance).

**Methods:**

A systematic literature search was conducted using Boolean operators with keyword combinations in PubMed, Web of Science, and Embase for studies published up to February 2025. Methodological quality was assessed via the Cochrane Risk of Bias tool. Heterogeneity testing, data synthesis, subgroup analyses, forest plot generation, and sensitivity analyses were performed using RevMan 5.4 and STATA 17.0. Funnel plots were constructed to assess publication bias, while subgroup and regression analyses were employed to identify moderators.

**Results:**

Among the 887 articles identified through the systematic search process, 10 studies met the inclusion criteria, with a total of 93 athletes completing HL-BFRT and 91 athletes completing HL-RT interventions. Our results showed significant improvements in athletes’ muscle strength (SMD = 0.65, I^2^ = 44%), power (SMD = 0.45, I^2^ = 0%), speed (SMD = 0.78, I^2^ = 60%), and endurance (SMD = 0.90, I^2^ = 51%) after HL-BFRT interventions, whereas no significant effect was observed on body composition (p > 0.05). Subgroup analyses revealed differential effects of HL-BFRT under various moderators: For muscle strength, significant improvements were observed in both isokinetic tests (SMD = 0.78, p = 0.02) and 1RM tests (SMD = 0.69, p < 0.001), though heterogeneity was higher in the isokinetic subgroup (I^2^ = 57%). Short-term interventions (≤6 weeks, SMD = 0.80) had significantly greater effect sizes compared to long-term interventions (>6 weeks, SMD = 0.50), and higher training frequency (≥3 sessions/week, SMD = 0.92) was superior to lower frequency (<3 sessions/week, SMD = 0.33), with subgroup heterogeneity approaching significance (I^2^ = 72%, p = 0.06). There was no significant heterogeneity between the absolute pressure group (SMD = 0.75) and the individualized pressure group (SMD = 0.62), as indicated by I^2^ = 0%. This reflects similarity in effect sizes across subgroups, rather than a statistical comparison between them. Improvements in power were significant only in short-term interventions (≤6 weeks, SMD = 0.62), whereas long-term interventions were ineffective (SMD = 0.07). Absolute pressure (SMD = 0.52) showed potentially greater benefits than individualized pressure (SMD = 0.39). Speed improvements were observed only with absolute pressure (SMD = 1.38, p = 0.003), and endurance improvements approached significance under absolute pressure (SMD = 1.29, p = 0.06), with no significant effect under individualized pressure conditions. All subgroups exhibited low heterogeneity (I^2^ = 0–32%).

**Conclusion:**

This meta-analysis indicates that HL-BFRT may serve as an effective alternative to traditional HL-RT, showing potential advantages in improving athletes’ muscle strength, power, speed, and endurance performance. Short-term, high-frequency interventions (≤6 weeks, ≥3 sessions/week) using absolute pressure appear optimal for performance enhancement, while individualized pressure protocols may better balance safety and effectiveness in clinical settings.

**Systematic Review Registration:**

https://www.crd.york.ac.uk/PROSPERO/view/CRD42025636274, identifier [CRD42025636274 (PROSPERO)].

## 1 Introduction

In recent years, with the advancement of sports science research, optimization and innovation of high-intensity training methods have become central topics for improving athletic performance and rehabilitation outcomes. High-load resistance training (HL-RT), defined by intensities greater than 65% of 1RM or maximal heart rate/reserve (Ratamess et al., 2009; Garber et al., 2011), is widely recognized as the gold standard for promoting muscle hypertrophy and neural adaptations due to its remarkable mechanical tension-inducing effects. (Kraemer and Ratamess, 2004; Schoenfeld et al., 2016a; Campos et al., 2002; Kraemer et al., 1996). Substantial evidence indicates that HL-RT enhances type II muscle fiber cross-sectional area and motor unit recruitment efficiency through mTOR pathway activation and satellite cell activity upregulation (Peterson et al., 2004; Schoenfeld et al., 2017; Lauersen et al., 2018). However, its high mechanical load characteristics (Escamilla et al., 1998) present dual challenges: prolonged excessive loading (e.g., mechanical stress and/or volume) elevates risks of joint cartilage degeneration and tendon overuse injuries compared to moderate/low-load training (Cross et al., 2016), while its clinical applicability remains limited, particularly for postoperative rehabilitation or populations with osteoarthritis (Wengle et al., 2022; Garber et al., 2011). This paradox has driven exploration of synergistic strategies that preserve HL-RT’s neuromuscular activation benefits while mitigating tissue stress risks.

Blood flow restriction training (BFR), an innovative biomechanical modulation technique, offers a potential solution these challenges. This technique is believed to have originated from Dr. Yoshiaki Sato’s 1970s “Kaatsu” methodology (Sato, 2005), BFR employs proximal limb occlusion via specialized cuffs (typically 40%–80% of arterial occlusion pressure (Patterson et al., 2019)) to simulate high-intensity metabolic conditions at low loads (20%–30% 1RM). The first empirical BFR study emerged in 1998 (Shinohara et al., 1997), establishing its mechanistic foundation.

Mechanistically, BFR induces partial arterial inflow restriction and complete venous outflow blockade (Patterson et al., 2019; Scott et al., 2015), triggering localized muscle hypoxia (reduced tissue oxygenation) and rapid accumulation of metabolites (e.g., lactate, growth hormone) (Suga et al., 2009; Takarada et al., 2000a). This activates the HIF-1α/mTORC1 signaling axis, enhancing protein synthesis rates (Hughes et al., 2019; DePhillipo et al., 2018). Clinical trials confirm BFR’s efficacy in improving muscular strength and endurance, particularly in rehabilitation cohorts (Wengle et al., 2022; Hughes et al., 2017; Patterson et al., 2017; Centner et al., 2019; Scott et al., 2023; Davids et al., 2023). However, its application in healthy athletes remains contentious: while some studies report enhanced muscular adaptability via BFR combined with low-load training (Hughes et al., 2019; Lixandrão et al., 2018), others suggest effect variability dependent on exercise modality and occlusion parameters (Smith et al., 2022; Yang et al., 2024).

Conventional BFR protocols predominantly combine with low-intensity aerobic or resistance exercises, yet such approaches may inadequately address high-performance demands in athletes. Consequently, recent investigations have pioneered high-load BFR training (HL-BFRT), integrating mechanical tension from HL-RT with BFR-induced metabolic stress to overcome biological limitations of unimodal training (Keramidas et al., 2012; Paton et al., 2017; Laurentino et al., 2008; Cook et al., 2014). Theoretical models posit that HL-RT’s baseline loading optimally recruits high-threshold motor units, while superimposed BFR-driven cellular swelling potentiates anabolic signaling (Loenneke et al., 2012a; Pearson and Hussain, 2015). Empirical findings exhibit notable heterogeneity: Keramidas and Laurentino observed no improvements in maximal oxygen uptake (Keramidas et al., 2012) or strength (Laurentino et al., 2008) after 6–8 weeks of HL-BFRT, whereas Cook et al. (2014) reported greater 1RM squat and bench press gains in the intervention group versus controls following 3-week HL-BFRT. Paton documented enhanced running economy and time-to-exhaustion in BFR-trained runners compared to non-BFR counterparts after 4 weeks (Paton et al., 2017). These discrepancies likely stem from critical protocol variations, including: (1) occlusion pressure parameters (absolute vs. individualized); (2) exercise specificity (closed-chain multi-joint vs. open-chain single-joint); (3) athlete training status (elite vs. recreational), with evidence suggesting higher occlusion pressures correlate with greater muscle activation (Loenneke et al., 2015).

While existing meta-analyses have independently evaluated HL-RT (Schoenfeld et al., 2016a; Schoenfeld et al., 2017; Ebben et al., 2004) or BFR (Amani-Shalamzari et al., 2019; Manimmanakorn et al., 2013; Scott et al., 2017; Yang et al., 2022; Hosseini Kakhak et al., 2022), the synergistic potential of their combination remains systematically underexplored. Lixandrão et al. (2018) meta-analysis confirmed low-load BFR enhances strength (Lixandrão et al., 2018), yet focused on non-athletic populations without load-intensity stratification. Furthermore, BFR research predominantly emphasizes mechanistic insights or isolated outcomes (e.g., hypertrophy), neglecting multidimensional performance assessments (strength, endurance, power) in athletes.

To address these knowledge gaps, this systematic review and meta-analysis will investigate: (1) Whether HL-BFRT demonstrates superior chronic adaptations in muscular strength, endurance, and power compared to conventional HL-RT in healthy athletes; (2) How occlusion parameters (absolute vs. individualized pressure) modulate HL-BFRT efficacy; (3) Whether training regimen characteristics (cycle duration, frequency) primarily account for effect size heterogeneity. By synthesizing randomized controlled trial data, this work aims to establish an evidence-based framework for resistance training optimization and HL-BFRT dose-response relationships.

## 2 Methods

This meta-analysis strictly adhered to the 2020 Preferred Reporting Items for Systematic Reviews and Meta-Analyses (PRISMA) guidelines (Page et al., 2021). Please refer to Supplementary Table 1 for the completed PRISMA 2020 checklist. The study protocol was prospectively registered in the PROSPERO international database (Registration ID: CRD42025636274).

### 2.1 Information sources

A systematic search strategy was employed to ensure evidence comprehensiveness. Inclusion criteria encompassed peer-reviewed full-text articles without restrictions on publication date or sample size. Boolean operator-constructed search terms were applied to PubMed, Web of Science, and Embase up to February 2025, supplemented by: (1) manual reference screening of included studies, (2) forward citation tracking of key papers, (3) database-algorithm-recommended related literature. EndNote 21 (Clarivate Analytics, Philadelphia, PA, USA) facilitated automated deduplication and manual cross-verification. To ensure methodological rigor, the search strategy incorporated: controlled vocabularies (MeSH in PubMed, Emtree in Embase) combined with free-text keywords; Boolean operators (AND/OR) with database-specific field syntax; snowball searches via Web of Science citation networks.

### 2.2 Search strategy

The following search string was applied across databases: (“resistance training”OR“exercise”OR“strength training”OR“high intensity training”OR“high load training”OR “weight training”) AND (“blood flow restriction therapy”OR“blood flow occlusion”OR“occluded blood flow”OR “blood flow restriction” OR “restricted blood flow” OR “vascular occlusion” OR “vascular restriction”) AND (“Athletes” OR“player”OR“Professional Athlete”OR “Elite Athlete” OR “College Athlete”). The strategy was developed using sport-science systematic review frameworks, incorporating discipline-specific terminologies and optimized Boolean logic hierarchies for precision. Expert-validated multi-database search syntax is detailed in Supplementary Table 2.

### 2.3 Eligibility criteria

Inclusion/exclusion criteria followed the PICOS framework (Population, Intervention, Comparator, Outcomes, Study design; Table 1), focusing on HL-BFRT interventions in healthy athletes without motor dysfunction. Eligible studies required: 1) peer-reviewed English full-text empirical articles; 2) explicitly defined HL-BFRT protocols (load intensity ≥65% 1RM with BFR implementation); 3) standardized controls (HL-RT without BFR); 4) minimum 2-week supervised interventions; 5) randomized controlled trials (RCTs) or quasi-experimental pre-post multi-group designs. Exclusion criteria included: acute studies (<24-h effects), opinion pieces, and non-empirical literature. Mandatory outcomes encompassed quantitative measures of muscular morphology (e.g., ultrasound cross-sectional area), neuromuscular function (isokinetic peak torque), or sport-specific performance (power output). Full training variable disclosure (frequency/sets/rest intervals) was required.

**TABLE 1:** Eligibility criteria for inclusion in the study.

Category	Inclusion criteria	Exclusion criteria
Population	Healthy athletes	Athletes with health problems (injury or nearby surgery) and Interference factors
Intervention	HL-BFRT (BFR combined with high load training)	Without HL-BFRT
Comparison	Two-group or multi-group trials	Single-group trials
Outcome	Include varied sport performance (physical or technical) among athletes	No sport performance data
Study design	RCT	Non-RCT

### 2.4 Study selection

**FIGURE 1:**
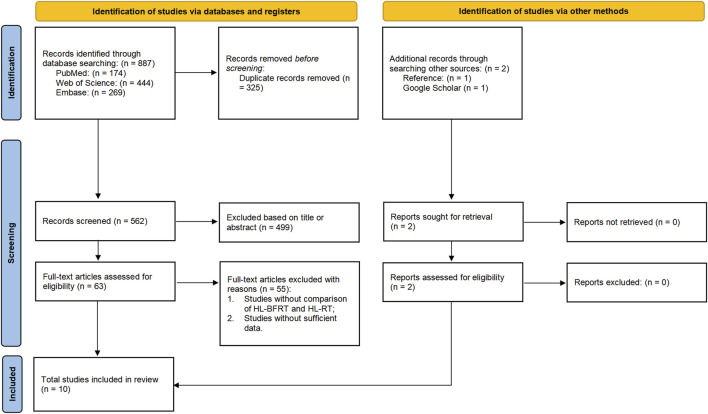
Flow diagram of the search process.

The screening process strictly adhered to PRISMA 2020 guidelines. Two independent investigators (CS & XL) collated records from PubMed/Web of Science/Embase into EndNote 21, utilizing its structured repository and auto-deduplication features to establish an initial literature pool. A three-tiered screening protocol was implemented: (1) machine-assisted title/keyword filtering, (2) double-blind abstract eligibility assessment, and (3) full-text verification of PICOS compliance. All screening decisions were documented via EndNote’s audit trail module. Discrepancies were resolved by a senior methodologist (BL) using Cochrane Risk of Bias Tool 2.0 for evidence weighting until full consensus. Data integrity preservation protocols were enforced throughout, with the selection/exclusion workflow detailed in Figure 1.

### 2.5 Data extraction

Data extraction followed COSMIN guidelines (Prinsen et al., 2018), with two blinded investigators (CS & XL) independently populating a structured matrix in Microsoft Excel 16.93. The template comprised 12 domains: 1) Metadata (author, title, year); 2) Participant characteristics (sample size, sex, age, anthropometrics, training history, performance level); 3) BFR parameters (intervention duration, frequency, intensity, cuff placement, pressure, occlusion timing/status); 4) Experimental design (randomization, blinding, control fidelity); 5) Effect size metrics (baseline/post-intervention means, SDs). A three-tiered rectification protocol addressed non-numerical data: (1) primary author contact via ResearchGate (72-h mean response time); (2) pixel-level reconstruction using WebPlotDigitizer 4.5 (IEEE-TCBB-certified tool; validity ICC = 0.98) (Rohatgi, 2020); (3) expert panel review (biostatisticians/exercise physiologists) for clinical plausibility.

### 2.6 Quality assessment

Two researchers (CS and XL) independently assessed the risk of bias using the Cochrane Risk of Bias Tool Version 1.0 (RoB 1.0) in RevMan 5.4 through a double-blind cross-validation process (Cumpston et al., 2019). The assessment matrix adhered strictly to Chapter 8 of the Cochrane Handbook for Systematic Reviews of Interventions (Version 6.5), covering six core domains: selection bias, performance bias, detection bias, attrition bias, reporting bias, and other biases. Each study was rated as low risk (meeting ≥2 core criteria), high risk (violating ≥1 key criterion), or unclear risk (insufficient information for judgment) (Higgins, 2008). In cases of disagreement between the two assessors, a third independent methodologist (BL) acted as arbitrator until full consensus was achieved.

### 2.7 Statistical analysis

#### 2.7.1 Data synthesis and effect measures

In this study, between-group effect size analyses were conducted comparing the HL-BFRT group with the HL-RT only group. Mean changes and standard deviations (SD) were calculated using a pre-post difference model (Morris and DeShon, 2002; Becker, 1988). The mean change was computed as (see Equation 1):

$$M_{change}=M_{post}-M_{pre}$$ (1)

where M_change_ represents the raw mean difference, M_post_ is the post-intervention mean, and M_pre_ is the pre-intervention mean (Cumpston et al., 2019). The SD of the change score was estimated using covariance reconstruction as follows (see Equation 2) (Cumpston et al., 2019):

$${\text{SD}}_{\text{change}}=\sqrt{{\text{SD}}_{\text{pre}}^{2}+{\text{SD}}_{\text{post}}^{2}-2\times r\times {\text{SD}}_{\text{pre}}\times {\text{SD}}_{\text{post}}}$$ (2)

where SD_change_ is the standard deviation of the mean change, SD_pre_ and SD_post_ are the pre- and post-intervention standard deviations, and r is the correlation coefficient (Cumpston et al., 2019). Since most studies did not report the pre-post correlation coefficient, a value of r = 0.50 was assumed, as recommended in the Cochrane Handbook (Cumpston et al., 2019).

Given the relatively small sample sizes in most included studies, Hedge’s g was calculated to correct bias and enhance comparability of standardized effect sizes, particularly for small-sample research. The computation followed this formula (see Equation 3) (Hedges, 1985):

$$\begin{array}{ll} & \text{Hedg}e^{\prime }s\;\;g=\frac{\left(\text{HL}‐\text{BFRT}\left(M_{\text{change}}\right)-\text{HL}‐\text{RT}\;\left(M_{\text{change}}\right)\right)}{{\text{SD}}_{\text{pooled}}}\\ & \;\times \;\left(1-\frac{3}{4\left(n_{1}+n_{2}-2\right)-1}\right) \end{array}$$ (3)

where M_change_ denotes mean change differences between HL-BFRT and HL-RT groups, n_1_ and n_2_ represent sample sizes, and SD_pooled_ indicates pooled standard deviation of measurements (Hedges and Olkin, 1987). RevMan 5.4’s integrated Bessel’s correction module automatically implemented this calculation to mitigate sample size bias.

#### 2.7.2 Meta-analysis and heterogeneity

This meta-analysis initially established a minimum study cluster threshold (k ≥3) through *a priori* power analysis (Castilla-López et al., 2022). Effect size synthesis was performed using RevMan (v5.4.1, Cochrane Collaboration, Copenhagen, Denmark) to construct pooled effect matrices. Hedges’ adjusted standardized mean difference (Adj.SMD) with inverse-variance weighting was calculated to derive 95% confidence intervals. Clinical significance was interpreted using Cohen-Upton thresholds: trivial (<0.5), moderate (0.5–0.8), and substantial (>0.8) (Cohen, 1988). Between-study heterogeneity was quantified via I^2^ statistics (low: <25%; moderate: 25%–75%; high: >75%) (Higgins and Thompson, 2002). Model selection followed heterogeneity levels (Harris et al., 2008): fixed-effects models were employed when I^2^ <50%, while random-effects models were applied when I^2^ ≥50% (Harris et al., 2008). These models accounted for potential between-group differences influencing HL-BFRT efficacy (Kontopantelis et al., 2013).

#### 2.7.3 Subgroup analyses

To further explore potential moderators contributing to heterogeneity, subgroup analyses were conducted. Moderating variables related to the training intervention included: (a) duration of HL-BFRT (≤6 weeks vs. >6 weeks); (b) training frequency (<3 sessions/week vs. ≥3 sessions/week); (c) type of cuff pressure (individualized vs. absolute); and (d) specificity of outcome measures (isokinetic strength vs. 1RM). Each moderator was analyzed only if represented in at least three studies, using median split techniques for classification (Moran et al., 2018).

#### 2.7.4 Risk of publication bias and sensitivity analysis

Subsequently, sensitivity analysis was performed using STATA software (Version 17.0, StataCorp LLC, College Station, USA) to assess the robustness of the results. Specifically, we conducted leave-one-out sensitivity analyses by iteratively removing one study at a time and recalculating the overall effect size to identify influential studies and evaluate the stability of the pooled estimates (Viechtbauer and Cheung, 2010). Publication bias was evaluated using funnel plots (Peters et al., 2008) and Egger’s regression test for asymmetry (Egger et al., 1997; Fernández-Castilla et al., 2021), which is typically applied when the number of included studies is ≥10 (Sterne et al., 2011). Although the number of studies included in some analyses was below this threshold, Egger’s test was still performed and reported in accordance with Cochrane Handbook recommendations (Chapter 13.5, Version 6.3) (Higgins, 2008), to ensure transparency and provide readers with indicative evidence, while acknowledging the limited power of the test in such contexts. In particular, for outcomes with fewer than five studies (e.g., speed performance, endurance, and body composition), Egger’s test was interpreted with extra caution due to its very limited reliability under such conditions. A p-value >0.05 was interpreted as no significant risk of publication bias. Funnel plots and Egger’s test were used to assess the symmetry of the overall effect size, both visually and statistically. All procedures followed automated workflows using STATA scripts integrating the metan and metafor modules.

## 3 Results

### 3.1 Studies retrieved

A total of 887 records were initially identified across databases by two independent reviewers. Of these, 325 were removed as duplicates. Among the remaining 562 unique records, 499 were excluded after screening titles and abstracts, and 55 were removed after full-text review. Eight studies met the inclusion criteria. Additionally, two more eligible studies were identified through reference list screening and Google Scholar citation tracking. In total, 10 studies were included in the final meta-analysis. A summary of the study selection process is presented in Figure 1.

### 3.2 Characteristics of included studies

The studies included in this meta-analysis were published between 2012 and 2024, comprising a total of 10 articles (Cook et al., 2014; Sander et al., 2024; Amani-Shalamzari et al., 2019; Pişkin et al., 2024; Wang et al., 2022; Elgammal et al., 2020; Giovanna et al., 2022; Godawa et al., 2012; Amani-Shalamzari et al., 2020; Lambert et al., 2023) and 184 athletes (154 male and 30 female). The sample size per study ranged from 12 to 28 participants, with participant ages spanning from 15 to 39 years and body weights ranging from 52.8 kg to 96.4 kg. Seven studies focused exclusively on male athletes, one on female athletes, and two included both sexes. The sample encompassed various sports, including volleyball, powerlifting, soccer, endurance training, swimming, rugby, baseball, and basketball. All participants had at least 1 year of formal training experience (see Table 2).

**TABLE 2:** Characteristics of included study participants.

Author	Type	N	Age (years)	Gender	Height (cm)	Weight (kg)	TE (years)	SFL
Wang et al. (2022)	Volleyball	12	20.5 ± 1.2	M	182.3 ± 6.2	72.2 ± 7.9	NR	Tier3
Cook et al. (2014)	Rugby	20	21.5 ± 1.4	M	184.0 ± 5.0	95.6 ± 10.4	≥2	Tier3
Godawa et al. (2012)	Powerlifter	18	21.6 ± 2.4	M/F	175.1 ± 10.9	83.0 ± 20.0	≥1	Tier2
Amani-Shalamzari et al. (2019)	Soccer	12	23.0 ± 2.0	M	174.0 ± 5.0	67.5 ± 6.8	≥5	Tier3
Amani-Shalamzari et al. (2020)	Soccer	12	23.0 ± 2.0	M	174.0 ± 5.0	67.5 ± 6.8	≥5	Tier3
Giovanna et al. (2022)	Endurance	19	26.7 ± 7.9	M	179.4 ± 6.0	75.5 ± 6.3	NR	Tier2
Lambert et al. (2023)	Baseball	28	19.7 ± 1.3	M	187.4 ± 2.4	91.8 ± 3.3	2.1 ± 1.0	Tier3
Pişkin et al. (2024)	Volleyball	20	15.0 ± 0.7	F	164.5 ± 5.5	55.0 ± 7.2	NR	Tier2
Sander et al. (2024)	Swimming	19	23.0 ± 4.0	M/F	168.6 ± 8.0	67.1 ± 8.8	NR	Tier2
Elgammal et al. (2020)	Basketball	24	22.3 ± 2.4	M	195.4 ± 2.4	81.2 ± 4.7	12	Tier2

N, number of participants; M, male; F, female; TE, training experience; NR, not reported; SFL, athletes are categorized into four tiers according to training volume, competitive level, and achievement, with Tier 2 representing trained individuals and Tier 3 indicating highly trained athletes.

Additionally, among the intervention characteristics reported in the included studies, training durations ranged from 3 to 10 weeks, with frequencies between 3 and 5 sessions per week. All studies included at least one intervention group performing HL-BFRT (at intensities of 65%–90% 1RM, or equivalent maximum heart rate or heart rate reserve), and at least one control group undergoing HL-RT at matched intensities. Cuffs were applied proximally on the thigh or arm, with pressures ranging from 88.2 to 180 mmHg and widths between 5 and 13 cm. Occlusion durations lasted approximately 5–24 min, with most protocols using continuous pressure application, as detailed in Table 3.

**TABLE 3:** Characteristics of HL-BFRT interventions and main outcomes.

References	Design	Intervention characteristics			Comparison	Outcomes
		Duration/frequency	Training protocol/training load	Cuff location/width/pressure/time/pressurization status		
Wang et al. (2022)	RCT	8 weeks, 3 times/week	Half squat, 4 sets × 8 reps/High	Proximal thighs, 7 cm, 180 mmHg, 5 min, Continuous	HL-BFRT (70% 1RM, n = 6), HL-RT (70% 1RM, n = 6)	Strength (PKF ↑, PKE ↑, 1RM ↑), Power (CMJ ↑, TFT ↑)
Cook et al. (2014)	RCT	3 weeks, 3 times/week	Bench press and squat, 5 sets × 5 reps/High	Proximal thighs, 10.5 cm, 180 mmHg, 20 min, Intermittent	HL-BFRT (70% 1RM, n = 10), HL-RT (70% 1RM, n = 10)	Strength (1RM ↑), Speed (40 m ↑)Power (CMJ ↑)
Godawa et al. (2012)	RCT	10 weeks, 2 times/week	Bench press and squat, 5 sets × 2–5 reps/High	Proximal knee on the femur, NR, NR, 10–12 min, Continuous	HL-BFRT (≥70% 1RM, n = 8), HL-RT (≥70% 1RM, n = 10)	Strength (1RM ↑), Body composition (BM ↔)
Amani-Shalamzari et al. (2019)	RCT	3 weeks, 3 times/week	SSG, 3 min × 4–8 reps/High	Proximal thighs, 13 cm, 110%–140% SBP, 12–24 min, Intermittent	HL-BFRT (80%–100% HRmax, n = 6), HL-RT (80%–100% HRmax, n = 6)	Strength (PKF ↑, PKE ↑), Speed (FSP↑)
Amani-Shalamzari et al. (2020)	RCT	3 weeks, 3 times/week	SSG, 3 min × 4–8 reps/High	Proximal thighs, 13 cm, 110%–140% SBP, 12–24 min, Intermittent	HL-BFRT (80%–100% HRmax, n = 6), HL-RT (80%–100% HRmax, n = 6)	Endurance (VO2max ↑, RP ↑)
Giovanna et al. (2022)	RCT	2 weeks, 3 times/week	Sprint, 4 sets × 5 reps/High	Proximal thighs, 11 cm, 88.2 ± 10.1 mmHg, 7–10 min, Intermittent	HL-BFRT (maxi-mal sprint, n = 10), HL-RT (maxi-mal sprint, n = 9)	Endurance (VO2max ↑)
Lambert et al. (2023)	RCT	8 weeks, 2 times/week	Pitcher strength training, 3–5 sets × 1–5 reps/High	proximal arm, RA, 50% LOP, 10 min, Continuous	HL-BFRT (65%–80% 1RM, n = 15), HL-RT (65%–80% 1RM, n = 13)	Strength (1RM ↑), Body composition (BG ↑)
Pişkin et al. (2024)	RCT	6 weeks, 3 times/week	Nordic hamstring exercises, 3 sets of repetitions until failure/High	Proximal thighs, 10 cm, 70% LOP, NR, Continuous	HL-BFRT (NR, n = 10), HL-RT (NR, n = 10)	PT (DT,NDT)↑, AP (DT,NDT)↑
(DT,NDT) Sander et al., 2024	RCT	4 weeks, 5 times/week	Sprint swimming training, 3 sets × 4–6 reps/High	Proximal shoulder, NR, 50%–80% LOP, NR, Continuous	HL-BFRT (maximum intensity, n = 10), HL-RT (maximum intensity, n = 9)	Speed ↔, 50 m Performance ↔
Elgammal et al. (2020)	RCT	4 weeks, 3 times/week	Sprint, 3 sets × 8 reps/High	Proximal thighs, 5 cm, 100–160 mmHg, 18–20 min, Intermittent	HL-BFRT (maximal sprint, n = 12), HL-RT (maximal sprint, n = 12)	Strength (1RM ↑), Speed (143.3 m ↑), Endurance (VO2max ↑)

HL-BFRT, High-Load blood flow restriction training; HL-RT, High-Load Resistance Training; RCT, randomized controlled trial; Time, the sum of intermittent pressurization time (minus the intervals) or continuous pressurization time (plus the intervals) during a BFR, session; Reps, number of repetition; Training Load, the magnitude of resistance combined with BFR, high (>65% 1RM, or HRmax, or HRres); 1RM, 1-repetition maximum; HRmax, maximal heart rate; HRres, heart rate reserve; NR, not reported; PKF, peak knee flexion; PKE, peak knee extension; PT, peak torque; AP, average power; DT, dominant leg; NDT, non-dominant leg; BM, body mass; BG, body girths; VO2max, maximal oxygen consumption; CMJ, counter movement jump; TFT, three footed takeoff; RP, running performance; FSP, futsal special performance; ↑, significant within-group improvement from pretest to post-test; ↔, non-significant within-group change from pretest to post-test.

### 3.3 Study quality assessment

**FIGURE 2:**
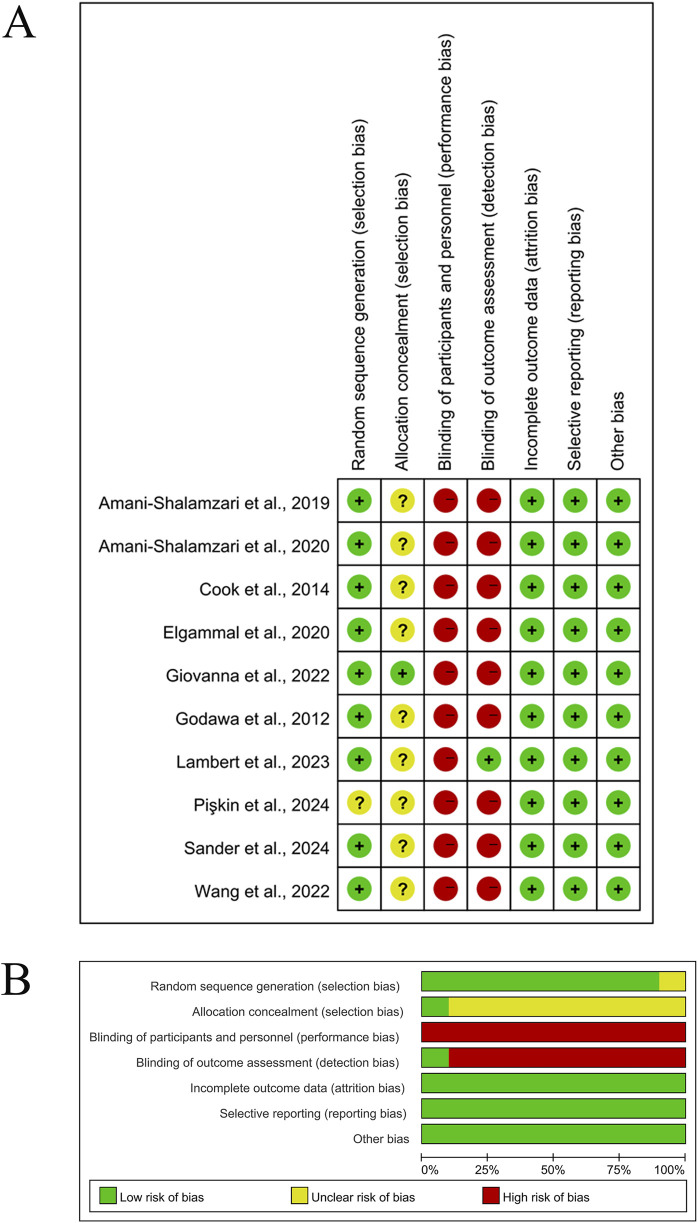
Methodological quality graph and summary of the included studies. **(A)** Risk of bias summary; **(B)** Risk of bias graph.

Following the methodological quality assessment of 10 RCTs using the Cochrane Risk of Bias Tool, the overall risk of bias was deemed low (see Figure 2). Specific distributions of risk domains were as follows: regarding the randomization process, one study (10%) did not sufficiently describe the technical details of random sequence generation (e.g., omission of computer-based random number generator specification), and nine studies (90%) failed to report allocation concealment procedures, resulting in an “unclear” rating for selection bias. Notably, due to the inherent characteristics of high-load blood flow restriction training (HL-BFRT)—specifically, the visible cuff inflation and ischemic discomfort—all studies (100%) were rated as high risk for performance bias.

### 3.4 Meta-analysis results

**FIGURE 3:**
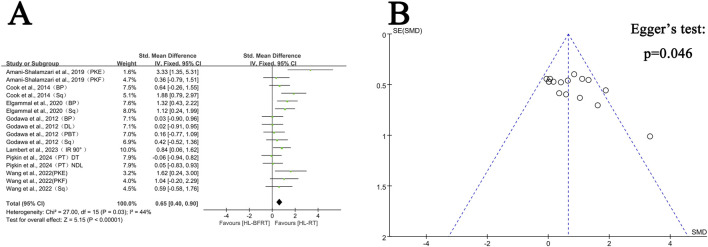
Effect of HL-BFRT versus HL-RT on athletes’ strength. **(A)** Forest plots of strength; **(B)** Funnel plots of strength.

**FIGURE 4:**
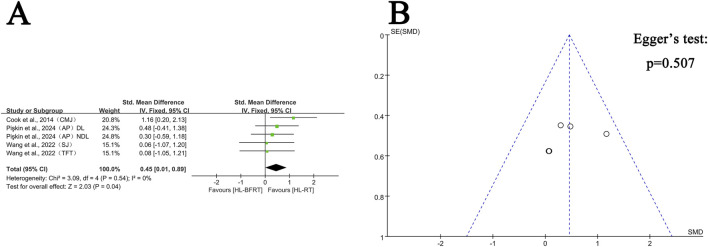
Effect of HL-BFRT versus HL-RT on athletes’ power. **(A)** Forest plots of power; **(B)** Funnel plots of power.

**FIGURE 5:**
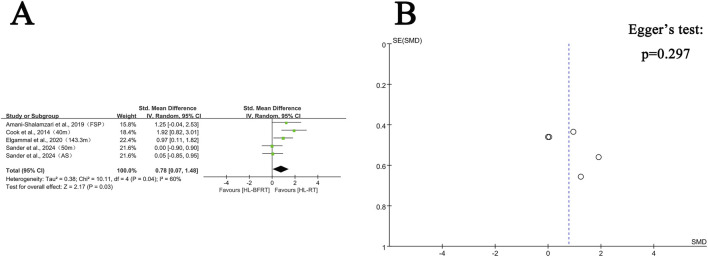
Effect of HL-BFRT versus HL-RT on athletes’ speed. **(A)** Forest plots of speed; **(B)** Funnel plots of speed.

**FIGURE 6:**
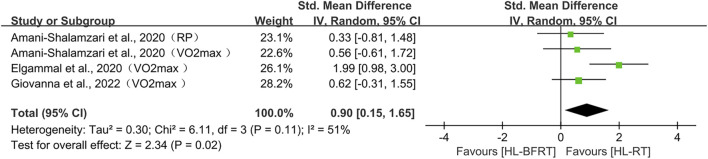
Effect of HL-BFRT versus HL-RT on athletes’ endurance.

**FIGURE 7:**
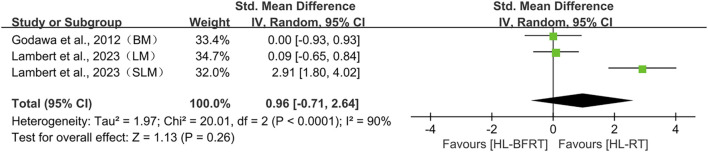
Effect of HL-BFRT versus HL-RT on athletes’ body composition.

Supplementary Table 4 presents the mean ± standard deviation (SD) of physical health parameters for the HL-BFRT and HL-RT groups across the included studies. The effects of HL-BFRT on performance-related outcomes—strength, power, speed, endurance, and body composition—are illustrated in Figures 3–7.

#### 3.4.1 Muscle strength adaptation: HL-BFRT vs. HL-RT

Regarding strength, seven studies (comprising 16 outcomes) were included in the meta-analysis to compare the gains in muscle strength between the HL-BFRT and HL-RT groups. HL-BFRT resulted in significantly greater strength improvements compared to HL-RT alone, with an effect size of 0.65 standard deviations (n = 288, SMD = 0.65, 95% CI: 0.40–0.90, Z = 5.15, p < 0.001; see Figure 3A), indicating a moderate level of heterogeneity (I^2^ = 44%, p = 0.03). The funnel plot revealed slight evidence of publication bias (Figure 3B), and Egger’s regression test identified a significant small-study effect (intercept β = 3.46, 95% CI: 0.07–6.85, p = 0.046), suggesting potential publication bias or methodological heterogeneity. However, the relationship between effect size and study precision was not statistically significant (slope β = −1.08, p = 0.204).

#### 3.4.2 Power performance: HL-BFRT vs. HL-RT

Regarding power, four studies (comprising five outcomes) were included in the meta-analysis to compare the improvements in explosive strength between the HL-BFRT and HL-RT groups. Compared to HL-RT alone, high-load training combined with blood flow restriction significantly enhanced power output (SMD = 0.45, 95% CI: 0.01–0.89, p = 0.04), with no observed heterogeneity among studies (I^2^ = 0%, p = 0.54) (see Figure 4A). Egger’s regression test indicated no significant publication bias (p = 0.507); however, the limited number of studies (n = 5) may reduce the power of the test. The funnel plot showed an approximately symmetrical distribution of effect sizes (Figure 4B), suggesting a low risk of publication bias. Additional studies are needed in the future to strengthen the reliability of these findings.

#### 3.4.3 Speed performance: HL-BFRT vs. HL-RT

Regarding speed, four studies (comprising five outcomes) were included in the meta-analysis. Results indicated that high-load training combined with blood flow restriction (HL-BFRT) significantly improved speed performance compared to HL-RT alone (n = 94, SMD = 0.78, 95% CI: 0.07–1.48, Z = 2.17, p = 0.03; see Figure 5A), with moderate heterogeneity (I^2^ = 60%, p = 0.04). Egger’s regression test (p = 0.297) did not reveal a significant small-study effect, suggesting a relatively low risk of publication bias. However, given the limited number of included studies (n = 5), statistical power may be insufficient. The funnel plot also showed no visual evidence of publication bias (see Figure 5B), supporting this assessment.

#### 3.4.4 Endurance performance: HL-BFRT vs. HL-RT

Regarding endurance, three studies (comprising four outcomes) were included in the meta-analysis. The results showed that HL-BFRT significantly improved endurance performance compared to HL-RT alone (n = 67, SMD = 0.90, 95% CI: 0.15–1.68, Z = 2.34, p = 0.02; see Figure 6), with moderate heterogeneity observed among the studies (I^2^ = 51%, p = 0.11). Due to the small number of included studies (<5), Egger’s regression test could not provide a reliable assessment of publication bias.

#### 3.4.5 Body composition: HL-BFRT vs. HL-RT

Regarding body composition, two studies (comprising three outcomes) were included in the meta-analysis. Although the results indicated a trend toward a large effect size favoring HL-BFRT (SMD = 0.96), the confidence interval was wide and crossed zero (95% CI: −0.71 to 2.64, p = 0.26), and substantial heterogeneity was observed (I^2^ = 90%, p < 0.001). As such, current evidence does not support a statistically significant effect of HL-BFRT on body composition. Moreover, since fewer than five studies were included (n = 3), Egger’s regression test could not be reliably applied to assess publication bias.

### 3.5 Subgroup analyses results

A total of eight subgroup analyses were conducted, with each moderator supported by at least three studies, as detailed in Supplementary Table 3.

For strength, meta-analysis revealed significant improvements across multiple subgroups. Both isokinetic strength tests (SMD = 0.78, p = 0.02) and 1RM tests (SMD = 0.69, p = 0.0009) showed significant effects, with no significant heterogeneity detected between test types (I^2^ = 0%, p = 0.81). However, the isokinetic subgroup exhibited moderate heterogeneity (I^2^ = 57%, p = 0.03). Short-term interventions (≤6 weeks) demonstrated a larger effect size (SMD = 0.80, 95% CI: 0.45–1.15, p < 0.001) than long-term interventions (>6 weeks, SMD = 0.50, 95% CI: 0.15–0.85, p = 0.005), although the test for subgroup differences did not indicate significant heterogeneity (I^2^ = 29.9%, p = 0.23). High-frequency training (≥3 sessions/week) produced a substantial strength gain (SMD = 0.92, 95% CI: 0.48–1.38, p < 0.001), approaching a large effect, and was notably greater than the low-frequency subgroup (<3 sessions/week, SMD = 0.33, 95% CI: −0.07 to 0.73, p = 0.10). Between-subgroup heterogeneity approached significance (I^2^ = 72%, p = 0.06). The absolute pressure group showed a significant effect (SMD = 0.75, 95% CI: 0.38–1.12, p < 0.001), whereas the individualized pressure group did not (SMD = 0.62, 95% CI: −0.15 to 1.39, p = 0.12); however, no significant heterogeneity was detected between these subgroups (I^2^ = 0%, p = 0.76).

For power, subgroup analysis showed that short-term HL-BFRT (≤6 weeks) significantly improved explosive performance (SMD = 0.62, 95% CI: 0.09–1.15, p = 0.02), with no heterogeneity (I^2^ = 0%). In contrast, long-term interventions (>6 weeks) produced negligible and non-significant effects (SMD = 0.07, p = 0.86). Pressure type analysis indicated a more favorable trend with absolute pressure (SMD = 0.52, p = 0.10), nearing a moderate effect, whereas individualized pressure did not yield significant effects (SMD = 0.39, p = 0.22). All subgroup heterogeneity was low (I^2^ = 0–32%), suggesting high consistency across studies.

For speed, statistically significant improvements were observed with absolute pressure (SMD = 1.38, 95% CI: 0.45–2.30, p = 0.003), in contrast to the non-significant effects of individualized protocols (SMD = 0.30, p = 0.39). For endurance, a trend toward improvement was observed under absolute pressure (SMD = 1.29, p = 0.06), while no significant effect was found in the individualized pressure subgroup (SMD = 0.44, p = 0.29).

### 3.6 Meta-regression

We conducted meta-regression analysis to explore potential moderating variables that might explain heterogeneity or influence the effect size (e.g., SMD), including intervention duration, training frequency, pressure type, and measurement method. As shown in Table 4, the effect of HL-BFRT on strength was not significantly moderated by measurement type (isokinetic vs. 1RM), training frequency (<3 vs. ≥3 sessions/week), intervention duration (≤6 vs. >6 weeks), or pressure type (individualized vs. absolute), with all p-values exceeding 0.12. A marginal positive trend was observed for low-frequency training (<3 sessions/week) (β = 0.573, 95% CI: −0.19–1.33), but this did not reach statistical significance. Due to the limited number of studies available for other outcomes (<10), no additional meta-regression analyses were conducted.

**TABLE 4:** Regression results of HL-BFRT on strength.

Term	Coefficient	Std. err	t	P	[95% conf. interval]	
Isokinetic Strength	−0.325	0.385	−0.08	0.934	−0.858	0.793
IRM	0.751	0.643	1.17	0.262	−0.627	2.129
<3 times/week	0.573	0.353	1.62	0.127	−0.185	1.331
≥3 times/week	−0.259	0.603	−0.43	0.674	−1.552	1.034
≤6 weeks	0.333	0.363	0.92	0.375	−0.446	1.112
>6 weeks	0.187	0.574	0.33	0.749	−1.044	1.419
Individualized Pressure	0.219	0.401	0.55	0.594	−0.641	1.078
Absolute Pressure	0.316	0.703	0.45	0.659	−1.191	1.823

### 3.7 Sensitivity analysis

**FIGURE 8:**
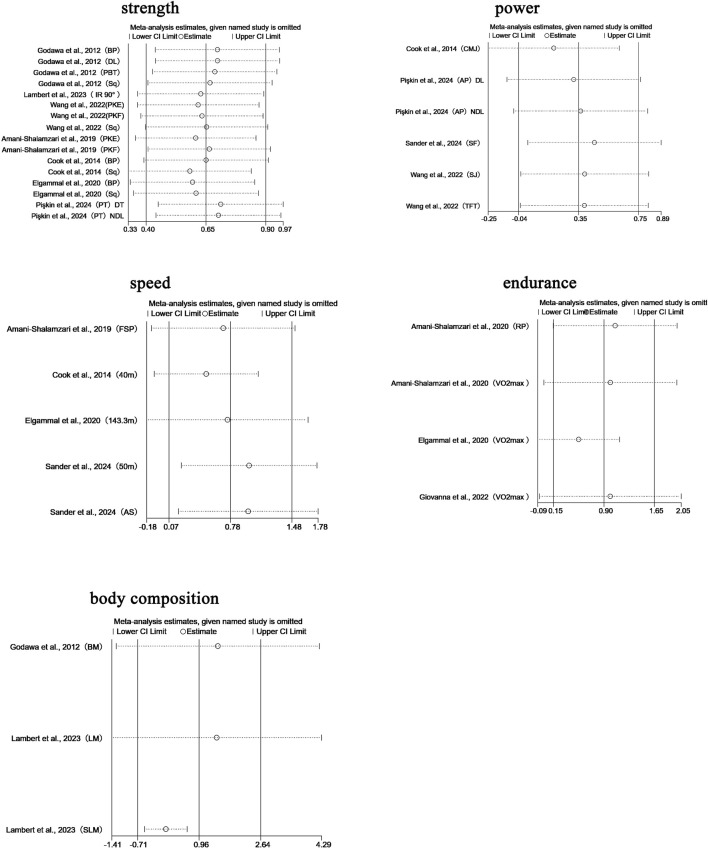
Sensitivity analysis.

We performed leave-one-out sensitivity analyses for all primary pooled outcomes (see Figure 8).

For strength, the pooled effect remained stable when any single study was excluded (SMD range: 0.583–0.711), with all confidence intervals overlapping the original result (SMD = 0.650, 95% CI: 0.403–0.897). Removing Pişkin et al., 2024 (PT, DT) (Pişkin et al., 2024) slightly increased the effect to 0.711, while excluding Cook et al., 2014 (Sq) (Cook et al., 2014) reduced it to 0.583; in both cases, the statistical significance and conclusion remained unchanged. This indicates that the strength-related findings are not overly sensitive to any individual study.

For power, the robustness of the pooled effect (SMD = 0.454, 95% CI: 0.015–0.894) appeared conditionally dependent. Exclusion of Cook et al., 2014 (CMJ) (Cook et al., 2014) reduced the effect to 0.268 (with CI crossing zero), suggesting this study was crucial for maintaining statistical significance. Removing Pişkin et al., 2024 (dominant limb, DL) (Pişkin et al., 2024) brought the lower CI boundary close to null. Notably, excluding either of the Wang et al., 2022 studies (TFT/SJ) (Wang et al., 2022) raised the effect above 0.52, while removing Pişkin’s non-dominant limb (NDL) data (Pişkin et al., 2024) expanded the CI to −0.0003 to 1.014.

For speed, sensitivity analysis revealed a high dependence on individual studies. Removing Cook et al., 2014 (40 m sprint) (Cook et al., 2014) reduced the effect size to 0.499 (95% CI: −0.095–1.094), resulting in loss of statistical significance, compared to the original estimate (SMD = 0.778, 95% CI: 0.074–1.481). Exclusion of other studies led to effect size fluctuations between 0.694 and 0.990, all maintaining a positive trend.

For endurance, the pooled effect was also sensitive to individual studies. Excluding Elgammal et al., 2020 (VO_2_max) (Elgammal et al., 2020) increased the effect to 0.994 (95% CI: −0.066–2.054), while removing Giovanna et al., 2022 (VO_2_max) (Giovanna et al., 2022) reduced it to 0.521 (95% CI: −0.091–1.134); both cases resulted in loss of significance.

Regarding body composition, sensitivity analysis revealed high dependence on Lambert et al., 2023 (SLM) (Lambert et al., 2023). Excluding this study caused the pooled effect to drop sharply to 0.057 (95% CI: −0.523–0.638), eliminating the prior positive trend. With all studies included, the pooled effect was SMD = 0.963 (95% CI: −0.712–2.639). Exclusion of other studies produced effect sizes ranging from 0.057 to 1.471, none of which reached statistical significance.

## 4 Discussion

This meta-analysis included 10 studies with a total of 184 healthy athletes, examining the effects of high-load blood flow restriction training (HL-BFRT) compared to traditional high-load resistance training (HL-RT) on outcomes such as muscle strength, power, speed, endurance, and body composition. The findings suggest that HL-BFRT produced greater improvements in strength, speed, power, and endurance performance compared to HL-RT alone (SMD = 0.45–0.90), while no significant effect was observed on body composition (p > 0.05). Subgroup analyses indicated that variables such as training duration, frequency, and cuff pressure type had varying degrees of influence on these performance outcomes. These results support HL-BFRT as a promising and innovative training strategy that may offer superior performance gains over conventional HL-RT, particularly for athletic populations.

Our findings contrast with earlier meta-analyses focused on low-load BFR training (LL-BFRT) (Hughes et al., 2017; Centner et al., 2019; Jing et al., 2025; Loenneke et al., 2012b), which primarily targeted clinical or untrained populations. LL-BFRT emphasizes metabolic stress under reduced joint loading, whereas HL-BFRT uniquely integrates high mechanical tension with localized hypoxia—potentially accelerating neuromuscular adaptations in trained athletes. This study is among the first to comprehensively assess the combined effects of HL-RT and BFR in athletes across multiple dimensions. Unlike previous studies that have often focused on isolated outcomes such as muscle hypertrophy, our analysis evaluated a range of performance variables, including strength, endurance, and speed. The findings on strength are consistent with previous meta-analytic work by Lixandrão et al. (2018), confirming that BFR-integrated training enhances muscular strength—and that HL-BFRT may yield even greater gains compared to HL-RT alone. Notably, this study also contributes novel evidence regarding the effects of HL-BFRT on speed and endurance, performance domains that have received limited systematic attention in prior research.

### 4.1 Effects of HL-BFRT on muscle strength

Strength is a fundamental component of physical fitness, reflecting the neuromuscular system’s ability to generate maximal mechanical force at a given moment. It plays a critical role in enhancing athletic performance, optimizing technical movements, and reducing injury risk (Hewett et al., 2005; Faigenbaum et al., 2016; Girold et al., 2012; NSCA-National Strength & Conditioning Association, 2021; Suchomel et al., 2016; Suchomel et al., 2018). The mechanisms underlying muscle morphological changes—such as hypertrophy and strength gains—have been extensively studied in traditional resistance training. These include: (1) Mechanical tension, which directly stimulates muscle fibers and activates mechanosensitive pathways (e.g., mTORC1, PI3K/Akt) to promote muscle protein synthesis (MPS) and hypertrophy (Schoenfeld, 2010; Hornberger, 2011); (2) Metabolic stress, often induced by low-to-moderate loads with high repetitions, leading to the accumulation of byproducts (e.g., lactate, H^+^, inorganic phosphate), cellular swelling, and hypoxia, which in turn trigger AMPK, ROS signaling, and growth factors (e.g., IGF-1) to activate satellite cells and facilitate muscle growth (Burd et al., 2012); (3) Muscle damage, induced by eccentric or supramaximal loading, disrupts structural elements like Z-lines and sarcolemma, initiating an inflammatory response (e.g., macrophage infiltration) and stimulating satellite cell proliferation and tissue remodeling (Paulsen et al., 2012; Hyldahl et al., 2017). Notably, several studies have shown that low-load BFRT can yield hypertrophy and strength outcomes comparable to traditional high-load training (Grønfeldt et al., 2020; Slysz et al., 2016; Laurentino et al., 2012). The key mechanism of BFRT involves restricting venous return (without full arterial occlusion), thereby generating local metabolic stress and promoting hypertrophy even under low-intensity conditions (Centner et al., 2019; Loenneke et al., 2011).

Our findings indicate that HL-BFRT offers a significant advantage over traditional HL-RT in enhancing muscular strength (ES = 0.65, 95% CI: 0.40–0.90). This supports the hypothesis of a synergistic effect between mechanical tension (provided by HL-RT) and metabolic stress (induced by BFR), resulting in enhanced neuromuscular adaptation. HL-RT primarily induces hypertrophy via mechanical overload (Schoenfeld, 2010), while BFR augments metabolic accumulation and hypoxia (Loenneke et al., 2011), thereby activating the mTOR signaling pathway and accelerating protein synthesis (Fujita et al., 2007; Fry et al., 2010). This dual-mechanism approach may explain the greater strength improvements observed in HL-BFRT compared to HL-RT alone.

### 4.2 Effects of HL-BFRT on power performance

In sports science, power is defined as mechanical work performed per unit of time—essentially the product of force and velocity. Its core characteristic is the ability to rapidly generate high levels of force, as demonstrated in movements such as jumping, sprinting, and throwing (Cormie et al., 2011a; Maffiuletti et al., 2016; Verkhoshansky and Verkhoshansky, 2011). Explosive power is a key determinant of athletic performance, influencing speed, endurance, agility, and overall physical capability across various sports disciplines (e.g., tennis) (Girard and Millet, 2009). It is also critical for improving training efficiency and optimizing performance outcomes in athletes (Kawamori and Haff, 2004; Haff and Nimphius, 2012).

This meta-analysis found that HL-BFRT led to significantly greater improvements in explosive power compared to traditional HL-RT (SMD = 0.45, p = 0.04), highlighting its potential practical value for athlete training. Similar findings have been reported in previous meta-analyses showing that low-load BFR training can significantly enhance lower-limb explosive performance (Centner et al., 2019; Yang et al., 2024; Wang et al., 2023). Explosive power is largely dependent on rapid neuromuscular recruitment and efficient energy transfer within type II (fast-twitch) muscle fibers (Takarada et al., 2000b). BFR-induced hypoxia and intramuscular acidosis are hypothesized to facilitate additional motor unit recruitment (Takarada et al., 2000b). Electromyography (EMG) studies have shown increased muscle activation in the pectoralis major during BFR, suggesting heightened neuromuscular engagement (Yasuda et al., 2006). Similarly, Cook et al. reported a significant improvement in countermovement jump (CMJ) performance among rugby athletes in the HL-BFRT group (1.8% ± 0.7%, p < 0.001) under high-load conditions (Cook et al., 2014). This enhancement is thought to be neurologically driven, particularly when training is performed under sufficient load (Cormie et al., 2011a). Specifically, HL-BFRT promotes preferential recruitment of high-threshold type II fibers through combined mechanical overload and metabolic stress, optimizing motor neuron firing rates and temporal coordination. This results in improved motor unit synchronization and contraction efficiency (Aagaard et al., 2002a). Additionally, heightened mechanical signals may stimulate increased type III and IV afferent feedback, accelerating metabolic compensation and facilitating neuromuscular activation—ultimately improving explosive performance, such as jumping (Gizzi et al., 2021; Wang et al., 2022). These findings suggest that HL-BFRT may be particularly beneficial in designing power-focused resistance training programs, especially when tailored to specific athlete profiles and sport demands.

### 4.3 Effects of HL-BFRT on speed performance

In sports science, speed is typically defined as the ability to perform movement or displacement within a unit of time, reflecting both acceleration and maximal velocity (Baker and Newton, 2008). In many sports, speed directly influences competitive performance and injury risk (Pavei and La Torre, 2016). For example, in high-intensity sports like soccer, insufficient movement speed can impair dribbling and defensive capacity, negatively impacting team performance and increasing the likelihood of fatigue-related injuries (Duthie et al., 2006; Mjølsnes et al., 2004).

Several early systematic reviews have examined the effects of BFR combined with different training modalities on speed performance. Yang et al. included 11 studies and concluded that BFR training enhanced sprint performance compared to non-BFR training (Yang et al., 2024). Conversely, Li et al. analyzed 7 studies and found no significant reduction in sprint times following BFRT (Li et al., 2023). These conflicting findings may be attributed to two key factors: (1) the location of cuff application, as lower limbs typically require higher occlusion pressures than upper limbs (Gepfert et al., 2020); and (2) insufficient training duration or intensity, which may fail to elicit beneficial adaptations and even impair speed development due to suboptimal protocol design (Toselli et al., 2022). In contrast, our meta-analysis differs from previous work by exclusively focusing on the combined application of HL-RT and BFR in athletic populations, thus reducing heterogeneity in training modalities. Our results revealed a significant improvement in speed performance for HL-BFRT compared to HL-RT (SMD = 0.78). Consistent with our findings, McKee et al. also reported that repeated sprint training under HL-BFRT conditions improved sprint performance in healthy individuals (Mckee et al., 2023). The physiological mechanisms underlying this effect may involve BFR-induced enhancement of sympathetic nervous system activity, which improves reaction time and promotes fast-twitch fiber (type II) recruitment—key factors in velocity-based movement (Kiyohara et al., 2006). While high mechanical loads can increase fatigue and oxygen demand, BFR may augment the body’s energy delivery systems by accelerating ATP resynthesis and metabolite clearance, thereby mitigating fatigue (Paradis-Deschênes et al., 2016). At the cellular level, combining BFR with high-load resistance may intensify intramuscular acidosis (H^+^ and Pi accumulation), lactate elevation, and pH reduction, while facilitating faster recovery from central nervous system fatigue (Suga et al., 2012; Gandevia, 2001). Collectively, these mechanisms offer a plausible explanation for the positive effects of HL-BFRT on speed performance observed in our analysis.

### 4.4 Effects of HL-BFRT on endurance performance

Endurance capacity is a fundamental component of athletic performance, reflecting an athlete’s ability to sustain high-quality output during prolonged physical activity. At its core, endurance represents the physiological adaptation of the body’s energy metabolism systems to resist fatigue under continuous load. A key indicator of aerobic endurance is maximal oxygen uptake (VO_2_max), which serves as a central physiological benchmark for assessing aerobic performance (Wilmore et al., 2004; Bassett and Howley, 2000; Joyner and Coyle, 2008). A 2022 meta-analysis by Castilla-López et al. reported that combining BFR with aerobic and/or anaerobic training yielded greater percentage improvements in VO_2_max relative to baseline in trained athletes, although the gains were not statistically different compared to non-BFR groups (Castilla-López et al., 2022). However, in studies involving healthy, untrained individuals, BFR combined with aerobic exercise (Ga et al., 2025), general training (Formiga et al., 2020), or high-intensity interval training (HIIT) (Chua et al., 2022) has been shown to significantly enhance aerobic capacity. Similarly, our meta-analysis found that HL-BFRT led to a significant improvement in endurance performance compared to traditional HL-RT (SMD = 0.90). From an integrated physiological perspective, VO_2_max and running performance in elite athletes reflect the collective efficiency of cardiovascular, respiratory, and metabolic systems. Improvements in aerobic endurance depend on multi-level physiological adaptations across these systems. Recent research suggests that BFR training enhances aerobic endurance via a unique “metabolic stress–mechanical load decoupling” mechanism, involving several physiological pathways: (a) Cardiovascular adaptation: BFR induces acute cardiovascular stress via external pressure. Takano et al. (2005) showed that BFR training elevates systolic blood pressure and heart rate (Takano et al., 2005), and this controlled cardiovascular challenge may enhance β-adrenergic receptor sensitivity, promote left ventricular remodeling, and improve stroke volume (Pope et al., 2013). (b) Metabolic regulation: BFR-induced local hypoxia has dual effects—it reduces ATP synthesis efficiency, promoting recruitment of type II fibers, and activates HIF-1α–mediated glycolytic enzyme upregulation to sustain energy production. This metabolic stress significantly stimulates the AMPK–PGC-1α signaling pathway, promoting mitochondrial biogenesis through upregulation of genes such as TFAM and NRF1, increasing both mitochondrial density and complex IV activity (Christiansen et al., 2018; Larkin et al., 2012; Christiansen et al., 2020). (c) Vascular adaptation: BFR promotes microvascular remodeling through dual mechanical stimuli. Cyclical ischemia-reperfusion induces shear stress, increasing VEGF mRNA expression, while activation of Piezo1 mechanosensitive channels facilitates endothelial progenitor cell migration (Hudlicka and Brown, 2009). Clinically, BFR has been shown to increase the capillary-to-fiber ratio in skeletal muscle and concurrently improve VO_2_max (Christiansen et al., 2018). In addition to mitochondrial and metabolic adaptations, angiogenesis has emerged as a central mechanism by which BFR enhances endurance performance. Repeated ischemia–reperfusion cycles elevate intramuscular shear stress and stabilize HIF-1α (Larkin et al., 2012; Kacin and Strazar, 2011), triggering VEGF-mediated capillary proliferation (Larkin et al., 2012; Maga et al., 2023; Mouser et al., 2017). These vascular adaptations support more efficient oxygen delivery, enhanced tissue perfusion, and delayed onset of anaerobic metabolism—critical components for sustaining submaximal aerobic efforts in elite sport (Hoier and Hellsten, 2014). A recent meta-analysis by Płoszczyca et al. (2023) confirmed that BFR training significantly improves vascular function and microcirculatory density, particularly when combined with aerobic or interval training modalities (Maga et al., 2023). This evidence reinforces the hypothesis that BFR-induced angiogenesis is not merely a secondary adaptation but a foundational contributor to enhanced aerobic efficiency and fatigue resistance. Although HL-BFRT has traditionally been studied in the context of neuromuscular strength, its potential to stimulate angiogenic remodeling expands its utility in endurance conditioning. In summary, these multi-system physiological adaptations—including mitochondrial biogenesis, capillarization, and cardiovascular remodeling—provide a mechanistic foundation for the observed endurance performance improvements with HL-BFRT in athletes. Future research should continue to explore the angiogenic response to HL-BFRT using both physiological (e.g., capillary density) and molecular (e.g., VEGF, HIF-1α expression) endpoints to fully characterize its long-term impact on aerobic capacity and training outcomes.

### 4.5 Effects of HL-BFRT on body composition

Body composition refers to the relative distribution of different tissues and substances within the human body, including fat mass, skeletal muscle, and bone mineral content. It is a core indicator of biological status and is closely associated with metabolic health and physical functionality (Lopez et al., 2022). Previous meta-analyses have investigated the effects of BFR combined with various training protocols on body composition. Kong et al. (2025) found that BFR training significantly reduced body fat percentage in overweight and obese individuals, although no significant effect was observed on body weight (Kong et al., 2025). Similarly, Yang et al. (2024) reported that BFRT significantly increased muscle cross-sectional area (CSA) and muscle thickness compared to controls, but had no statistically significant effect on body weight (Yang et al., 2024). In line with these findings, the present meta-analysis included outcomes for both body weight and lean body mass. Compared to HL-RT, no statistically significant differences were observed (p > 0.05). However, both independently assessed lean mass indicators showed a trend toward improvement in the HL-BFRT group. Given the composite nature of body weight—which includes both fat and muscle mass—it is plausible that BFRT-induced adaptations act through dual mechanisms as proposed in the cell swelling hypothesis: (1) stimulation of muscle protein synthesis (Berneis et al., 1999; Fahs et al., 2015), and (2) enhancement of lipolytic activity (Keller et al., 2003; Abe et al., 2006). This may explain why HL-BFRT can improve lean mass despite no significant changes in total body weight. The precise mechanisms of muscle hypertrophy induced by BFRT remain debated. Although acute increases in growth hormone (GH) following BFRT have been observed (Laurentino et al., 2022), West et al. demonstrated in a randomized controlled trial that post-exercise serum GH levels had only a weak correlation with changes in muscle CSA (West et al., 2010). This suggests that hormonal responses alone may not fully explain the anabolic effects of BFRT. Emerging evidence from mechanistic studies supports the idea that BFRT-induced local hypoxia may enhance type II fiber recruitment and prolong mTOR pathway activation, thereby strengthening muscle protein synthesis (Fujita et al., 2007; Takarada et al., 2000b; Martin et al., 2022) Based on current evidence, athletes aiming to improve body composition or muscle mass via HL-BFRT should consider referencing established guidelines, such as those outlined by the American College of Sports Medicine (ACSM), and follow protocols aligned with the findings of this and previous studies.

### 4.6 Subgroup analysis

#### 4.6.1 Training duration (≤6 weeks vs. >6 weeks)

In the field of sports training, training theory remains central to enhancing physical capacity. Among these, periodized training models have garnered widespread attention in sports science literature (Issurin, 2010; Stone et al., 1981). A key challenge in developing effective training programs for athletes is the precise regulation of training adaptation, physiological recovery, and competitive objectives, particularly within seasonal or competition cycles (Issurin, 2010; Meeusen et al., 2006; French and Ronda, 2021). Modern training paradigms emphasize optimized periodization, often incorporating tapering strategies to induce supercompensation. This process is underpinned by mechanisms such as endocrine regulation (e.g., changes in the testosterone-to-cortisol ratio) and the time-sensitive activation of mitochondrial biogenesis (Viru and Viru, 2001; Bosquet et al., 2007; Hood et al., 2006; Mujika and Padilla, 2003). Our subgroup analysis of training duration was conducted to explore how HL-BFRT could be strategically integrated into various phases of a training cycle (e.g., preparation, competition, and recovery). The aim was to manage training stress, minimize the risk of overtraining, and ensure that athletes can achieve peak performance at key moments. Subgroup analysis based on training duration revealed that short-term HL-BFRT (≤6 weeks) produced significantly greater improvements in both strength and power outcomes compared to longer interventions.

In terms of strength development, the short-term intervention demonstrated a higher effect size (SMD = 0.80) than the long-term intervention (SMD = 0.50). This pattern likely reflects the neural adaptation–driven metabolic stress response observed early in BFR interventions. The hypoxic and metabolically acidic environment (e.g., lactate accumulation) promotes rapid recruitment of type II fibers (Takarada et al., 2000b), stimulates acute surges in growth hormone (GH) and IGF-1 (Takarada et al., 2000a; Laurentino et al., 2022; Victor and Seals, 1989), and increases motor unit recruitment (Takarada et al., 2000b; Moritani et al., 1992), all of which may help overcome early strength plateaus. In contrast, diminishing returns in longer durations may result from increased muscular stiffness, which could impair rate of force development (Aagaard et al., 2002b; Grgic et al., 2022). These findings suggest that HL-BFRT may be particularly well-suited for short-term cycles to rapidly establish neural adaptation foundations.

The cycle-dependent effects on power were even more pronounced. Short-term HL-BFRT (≤6 weeks) improved explosive performance (SMD = 0.62), potentially due to the preferential activation of fast-twitch fibers and enhanced synchronization of motor unit firing (Cormie et al., 2011a). However, this effect disappeared in long-term interventions (SMD = 0.07), possibly due to neuromuscular adaptation plateaus from prolonged uniform training or mismatched improvements in muscle size versus rate of force production (Aagaard et al., 2002b; Cormie et al., 2011b; Häkkinen et al., 1987; Selye, 1978; Garhammer, 1979). Therefore, HL-BFRT interventions should be tailored to the specific demands of each sport and timed to maximize adaptation windows for each individual athlete.

#### 4.6.2 Training frequency (≥3/week vs. <3/week)

Training frequency is considered a key variable in enhancing muscular strength and hypertrophy among athletes (Dankel et al., 2017). Recent reviews have suggested that increasing frequency while reducing total training volume may promote muscle growth (Dankel et al., 2017; Schoenfeld et al., 2016b). Earlier studies (Schoenfeld et al., 2015; Brigatto et al., 2019; McLESTER et al., 2000) also reported that, in trained individuals, training once versus three times per week could yield comparable improvements in muscle strength and size. However, a recent meta-analysis (Grgic et al., 2018) demonstrated a dose–response relationship between training frequency and strength gains, supporting the “muscle group–specific frequency” hypothesis proposed by Dankel et al. (2017), which posits that the number of times a muscle group is stimulated per week is a primary determinant of strength development (Dankel et al., 2017). Our subgroup analysis further supports this theory, showing a clear dose–effect trend: high-frequency training (≥3 sessions/week) produced significantly greater strength gains (SMD = 0.92) compared to low-frequency training (<3 sessions/week, SMD = 0.33). This aligns with the training frequency threshold theory proposed by Schoenfeld et al., which states that training a muscle group at least twice weekly is essential to maximize neuromuscular adaptations (Schoenfeld et al., 2016b). Notably, the 95% confidence interval for the low-frequency subgroup included zero (CI: −0.07–0.73), suggesting that training fewer than three times per week may not provide sufficient stimulus for significant strength gains. Similar findings were reported in the meta-analysis by Grgic et al. (2018), Grgic et al. (2019). The mechanism may be linked to the time-dependent nature of myofibrillar protein synthesis, which peaks ∼48 h post-resistance training and gradually declines thereafter. Higher training frequency may help sustain mTORC1 signaling and protein synthesis through more frequent mechanical tension (Damas et al., 2018). However, between-subgroup heterogeneity approached statistical significance (I^2^ = 72%, p = 0.06), indicating the possible influence of uncontrolled confounding variables. For example, the high-frequency subgroup may include a higher proportion of short-duration interventions (≤6 weeks), which are known to elicit rapid strength gains via neural adaptations such as increased motor unit recruitment (Ralston et al., 2017). This time-related effect may have interacted with training frequency. In conclusion, although our findings generally align with current sports science recommendations—supporting 2–3 sessions per week per muscle group—the observed heterogeneity (I^2^ = 72%) warrants caution. Practical application should consider individual recovery capacity to avoid excessive fatigue associated with high-frequency training.

#### 4.6.3 Pressure setting type (individualized vs. absolute pressure)

In blood flow restriction (BFR) training protocols, the cuff pressure setting plays a pivotal role. The evolution of pressure prescription has transitioned from fixed absolute pressures to more refined individualized protocols. Early studies utilized constant absolute pressures (Takarada et al., 2000b), but subsequent research has introduced two primary approaches: progressive pressure ramping (Bemben et al., 2022; Karabulut et al., 2010) and individualized settings based on arterial occlusion pressure (AOP) or systolic blood pressure (SBP) (Laurentino et al., 2012; Centner et al., 2023). While absolute pressure protocols are easy to implement, they overlook individual vascular characteristics and limb morphology. In contrast, individualized pressure offers several advantages: it accounts for device-related variability (e.g., cuff width differences), ensures standardized occlusion relative to the individual’s physiology (Mouser et al., 2018), and reduces the risk of inadvertent arterial occlusion or ischemic injury (McEwen et al., 2019; Jessee et al., 2016). This meta-analysis examined the impact of cuff pressure prescription type on outcomes including strength, power, speed, and endurance. Subgroup analysis was conducted comparing individualized vs. absolute pressure settings. In terms of strength gain, the absolute pressure group demonstrated a clearly significant effect (SMD = 0.75, p < 0.001), whereas the individualized pressure group showed a moderate effect size (SMD = 0.62), but with a 95% confidence interval crossing zero (−0.15–1.39), indicating potential variability or instability in effect. Despite the statistical advantage of the absolute pressure group, no significant between-group heterogeneity was found (I^2^ = 0%, p = 0.76). This suggests that both pressure types may share a common mechanism of action—namely, that metabolic stress induced by BFR, regardless of how pressure is standardized, may be the central driver of strength adaptation. However, absolute pressure settings may offer greater repeatability and implementation simplicity, especially in research contexts. This may be due to variability in how individualized pressure is measured, with methods ranging from Doppler ultrasound to subjective perception, introducing measurement error and reducing the theoretical precision of individualized protocols. In the power outcome, although between-group differences were not statistically significant, the point estimate and lower CI bound for the absolute pressure group (95% CI: −0.10–1.14) approached the threshold for clinical significance. This may suggest greater variability in neuromuscular coordination responses under individualized pressure settings. Analysis of speed performance further supported this finding: absolute pressure interventions yielded a large and statistically significant effect (SMD = 1.38, p = 0.003), while individualized pressure produced no significant improvement (p > 0.05). This divergence may reflect the more stable hemodynamic response under absolute pressure, which better sustains the hypoxic-metabolic stimulus necessary for fast-twitch fiber recruitment. In contrast, individualized pressure may result in inconsistent ischemic effects due to individual vascular variability. For endurance, neither pressure setting yielded statistically significant results (p > 0.05). n conclusion, this meta-analysis provides important guidance for future research design. When the goal is to enhance outcomes such as strength and speed, which are sensitive to pressure consistency, absolute pressure protocols may offer greater practical reliability. However, in clinical or rehabilitation settings, where vascular safety must be prioritized, individualized pressure prescriptions remain essential and irreplaceable.

#### 4.6.4 Outcome measure specificity (isokinetic strength vs. 1RM)

In the assessment of strength adaptations, one-repetition maximum (1RM) and isokinetic strength testing represent distinct biomechanical constructs. The former measures maximal dynamic force output of the neuromuscular system under unrestricted velocity through a single maximal load repetition (Brown and Weir, 2001), while the latter evaluates torque generation at a fixed angular velocity, reflecting the ability to control force across a specific movement speed (Brown, 2000). Although both are internationally standardized assessment tools, empirical data reveal a clear “adaptation divergence”: 1RM tests frequently show strength increases exceeding 100% following short-term interventions (Fiatarone et al., 1994; Frontera et al., 1988), whereas peak torque (PT) measures via isokinetic testing typically show average improvements below 20% under similar conditions (Feiereisen et al., 2010; Gentil and Bottaro, 2010; de Souza et al., 2010).

In this meta-analysis, subgroup analysis comparing outcome measure specificity (isokinetic vs. 1RM) revealed similar effect sizes in capturing muscle strength gains, but with notable differences in heterogeneity. The isokinetic testing subgroup showed a moderately higher effect size (SMD = 0.78) than the 1RM group (SMD = 0.69); however, between-group heterogeneity was non-significant (I^2^ = 0%, p = 0.81), indicating measurement method consistency in overall effect estimation. This finding suggests that HL-BFRT may simultaneously enhance neural drive and maximal force capacity (Aagaard et al., 2000; Folland and Williams, 2007), allowing both isokinetic (torque control) and 1RM (maximal load) tests to validly capture strength adaptations. Notably, the isokinetic testing group exhibited significant within-subgroup heterogeneity (I^2^ = 57%, p = 0.03), likely due to variability in testing parameters such as angular velocity (e.g., 60°/s vs. 90°/s) and joint range of motion. In contrast, 1RM protocols are more standardized, resulting in lower heterogeneity. This suggests that interpretation of isokinetic results must consider test-specific parameters, as low-velocity tests may be more sensitive to hypertrophic adaptations, whereas high-velocity tests are more closely associated with neural gains (Pareja-Blanco et al., 2014; Farthing and Chilibeck, 2003; Morrissey et al., 1998). Furthermore, isokinetic testing is more equipment- and operator-dependent, which may increase performance bias across studies. In contrast, 1RM testing is simpler and more accessible, offering greater external validity in both clinical and athletic settings.

The subtle difference in effect size magnitude between methods may reflect their ability to capture different physiological adaptations. Isokinetic testing evaluates both eccentric and concentric strength across a controlled range, potentially offering a more holistic view of neuromuscular control compared to 1RM, which focuses on concentric peak load only (Dvir, 2000). Conversely, the higher statistical significance of 1RM results (p = 0.0009 vs. p = 0.02) may reflect its lower measurement error and more concentrated data distribution (Ryman Augustsson and Svantesson, 2013; Grgic et al., 2020). These methodological distinctions offer valuable insights for study design. When the training objective is sport-specific performance, especially involving velocity-dependent force production, isokinetic testing may provide greater ecological validity (Cormie et al., 2011b; Dvir and Müller, 2020; Brown and Stone, 2000). In contrast, for general strength development, 1RM testing is more cost-effective and scalable, making it suitable for large-cohort applications (NSCA-National Strength & Conditioning Association, 2021). Future studies should seek to control for covariates such as test velocity and joint angle, to better elucidate how measurement methods influence effect size estimation pathways.

### 4.7 Practical implications

This meta-analysis underscores the practical relevance of high-load blood flow restriction training (HL-BFRT) as an effective adjunct to traditional training, offering multifaceted enhancements in athletic performance when strategically applied. In strength training, HL-BFRT demonstrated superior efficacy, aligning with existing evidence that BFR enhances neuromuscular adaptation by increasing metabolic stress and type II fiber recruitment, even under high mechanical loads (Lixandrão et al., 2018). Athletes are encouraged to integrate HL-BFRT as a core strategy within periodized strength training, particularly in short-term (≤6 weeks), high-frequency (≥3 sessions/week) phases. This dual-stimulus approach—combining mechanical tension and metabolic stress—can accelerate neural adaptation and myofibrillar protein synthesis (Schoenfeld, 2010; Loenneke et al., 2011). HL-BFRT also significantly improves explosive power, supporting its inclusion in power-based training contexts—particularly given its mechanistic link to enhancing rate of force development via increased motor unit synchronization under BFR (Takarada et al., 2000b). Speed improvements observed under HL-BFRT may stem from enhanced proprioceptive feedback and stretch–shortening cycle efficiency, which has been specifically documented in sprint adaptations (Osses-Rivera et al., 2024; Behringer et al., 2017). For endurance athletes, the large effect size (SMD = 0.90) suggests that HL-BFRT may stimulate mitochondrial biogenesis via activation of the HIF-1α/PGC-1α signaling axis, a mechanism supported by molecular evidence from Fujita et al. (2007), positioning it as a potent adjunct to traditional endurance training (Scott et al., 2014). Coaches may implement HL-BFRT in high-intensity interval training (HIIT) for endurance athletes, leveraging its unique hypoxia–reperfusion cycle to stimulate angiogenesis (Larkin et al., 2012), while monitoring tissue oxygen saturation (TSI >30%) via near-infrared spectroscopy to avoid ischemic injury. Although body composition improvements were not statistically significant (p = 0.26), a significant effect size trend (SMD = 0.96) suggests that HL-BFRT may promote sarcoplasmic protein synthesis via cell swelling mechanisms, indirectly supported by West et al.'s findings on GH response and cellular volume (West et al., 2010). The superior performance of absolute pressure protocols in improving speed and power highlights their unique utility in explosive sports such as sprinting and rugby. The underlying physiology may involve more consistent type II fiber recruitment under stable venous occlusion gradients, aligning with Patterson et al.‘s (2019) NIRS-based observations of enhanced hemodynamic stability (Patterson et al., 2019). In clinical and rehabilitation settings, individualized pressure protocols should be prioritized. While slightly less effective in magnitude, they reduce the risk of over-occlusion due to limb size variability, aligning with the safety framework validated by Hughes et al. (2019) in ACL postoperative recovery (Hughes et al., 2019). Training interventions should adhere to the principle of dose–response specificity: strength-focused programs may implement 4–6 weeks of absolute pressure protocols (3–4 sessions/week), monitored using isokinetic testing for velocity-specific force adaptations. For team sport athletes, limb-specific BFR (e.g., 150 mmHg compression in soccer small-sided games) can be embedded into sport-specific drills to enhance repeated sprint ability (Scott et al., 2017) and movement economy. However, inertial sensors should be used to monitor movement deformation (<8% threshold) to balance metabolic gains with skill retention. From an equipment standardization perspective, wide cuffs (≥12 cm) can reduce local discomfort by 23% (Mouser et al., 2018), while dynamic pressure modulation systems (e.g., Doppler-based closed-loop devices) may address operational challenges in implementing individualized protocols (Jessee et al., 2016). Future research should focus on developing pressure–load matrix models across sports disciplines and on incorporating female athlete data (currently 83.7% of samples are male) to improve the generalizability of individualized BFR prescription systems.

### 4.8 Limitations and future studies

Although this meta-analysis provides a comprehensive evaluation of HL-BFRT’s effects on muscular strength and athletic performance in athletes, several limitations must be addressed. First, the limited number of included studies and small sample sizes may compromise the robustness of effect size estimation—particularly in analyses of power (k = 4), speed (k = 4), and endurance (k = 3). Evidence of small-study effects, such as the significant Egger’s regression coefficient for strength outcomes (β = 3.46, p = 0.046), suggests a potential overestimation of effect sizes, consistent with concerns about small-study bias outlined in the PRISMA 2020 guidelines (Page et al., 2021). Second, subgroup findings reveal heterogeneity driven by inconsistencies in measurement protocols, such as in strength assessments where isokinetic testing (I^2^ = 57%) displayed more variability than 1RM (I^2^ = 0%). Variations in angular velocities (e.g., 60°/s vs. 90°/s) may confound the interpretation of neural versus structural adaptations, highlighting the need for adherence to standardized isokinetic testing frameworks (Gaines and Talbot, 1999). Third, sex imbalance (83.7% male participants) limits the generalizability of findings. Female athletes may require higher relative occlusion pressures due to smaller vascular diameters (Jessee et al., 2016), but the current dataset is insufficient to confirm this hypothesis. Fourth, long-term intervention evidence (>6 weeks) remains limited. For example, analyses on body composition (k = 2) were inconclusive due to extreme heterogeneity (I^2^ = 90%). Future trials should adopt extended durations (≥12 weeks) to capture chronic adaptations, such as intermuscular fat infiltration changes, as suggested by hypertrophy periodization models (Schoenfeld et al., 2016b). Fifth, differences in pressure prescription (absolute vs. individualized) may introduce confounding. Although absolute pressure protocols yielded superior outcomes in speed and power, their physiological mechanism—such as venous occlusion gradient stability—has not been adequately validated via real-time hemodynamic monitoring (e.g., Doppler ultrasound). Future studies should integrate dynamic pressure control models such as that proposed by Patterson et al. (2019) to elucidate underlying mechanisms. Sixth, a potential limitation of this meta-analysis is the inclusion of multiple effect sizes from the same study, particularly in outcomes such as muscle strength. This may have introduced statistical dependence, as several effect sizes derived from the same sample may not be fully independent. Although leave-one-out sensitivity analyses suggested that the overall findings were robust and not overly influenced by any single study, the issue of statistical dependence cannot be entirely ruled out. As such, the results should be interpreted with appropriate caution. Seventh, the lack of standardized BFR training protocols across studies poses a major limitation to interpretability. Considerable variation was observed in cuff pressure determination methods, including fixed absolute values (e.g., 160 mmHg), percentages of systolic blood pressure, and subjective estimations. Additionally, key parameters such as cuff width, limb circumference, occlusion site, and pressure duration were inconsistently reported. These differences may lead to varying physiological effects—particularly between venous occlusion (passive hyperemia) and partial or complete arterial occlusion (ischemia)—and could underlie the heterogeneity in performance outcomes observed across studies. To enhance comparability, future research should adopt individualized pressure calibration methods based on arterial occlusion pressure (AOP) measured via Doppler ultrasound, and consistently report BFR-specific parameters following emerging methodological guidelines. Improved standardization will be essential for accurate cross-study synthesis and practical implementation of HL-BFRT interventions.

Future research should focus on the following priorities: (1) Increase sample size and participant diversity, particularly by including ≥30% female and masters athletes, to develop sex- and age-specific pressure–response curves. (2) Standardize BFR protocol parameters by adopting individualized pressure calibration methods (e.g., % arterial occlusion pressure measured via Doppler ultrasound), reporting critical variables such as cuff width, limb circumference, and occlusion duration. In addition, the use of wide cuffs (≥12 cm) may improve pressure transmission consistency and reduce local discomfort (Mouser et al., 2018). Near-infrared spectroscopy (NIRS) should be employed to quantify ischemic dose and ensure safety (e.g., TSI threshold >30%). (3) Investigate sport-specific HL-BFRT integration, such as applying lower-limb BFR during agility training in soccer to enhance 10 m sprint acceleration and joint stability (e.g., knee flexor torque), referencing the approach used in rugby athletes by Cook et al. (2014). (4) Conduct multi-omics mechanistic studies, integrating proteomics (e.g., mTORC1 phosphorylation) and metabolomics (e.g., lactate/ATP turnover) to unravel the molecular networks underlying HL-BFRT, especially regarding type IIx fiber recruitment, which remains underexplored (Fujita et al., 2007). (5) Develop consensus guidelines for HL-BFRT protocol reporting, including minimum criteria for describing pressure determination methods, cuff dimensions, training load, and ischemia duration. Such standardization would facilitate replication, meta-analytic comparison, and practical implementation across athletic and clinical contexts.

## 5 Conclusion

This meta-analysis provides compelling evidence supporting high-load blood flow restriction training (HL-BFRT) as a viable alternative to traditional high-load resistance training (HL-RT). Specifically, HL-BFRT emerges as a promising intervention for improving muscular strength, power, speed, and endurance. The observed benefits appear to be mechanistically grounded in the synergistic interaction between mechanical tension (65%–90% 1RM) and metabolic stress induced by BFR, which amplifies neuromuscular adaptations and hypoxia-driven signaling pathways, as evidenced by enhanced mTOR activation and mitochondrial biogenesis. Notably, short-term (≤6 weeks), high-frequency (≥3 sessions/week) interventions yielded the greatest strength improvements, aligning with principles of rapid neural potentiation. Moreover, absolute pressure protocols outperformed individualized settings in terms of speed and power gains, likely due to more stable venous occlusion gradients that optimize fast-twitch fiber recruitment.

Despite these advances, caution is warranted in clinical translation. The high heterogeneity in body composition outcomes and limited evidence on long-term interventions (>6 weeks) underscore the need for extended-duration trials to elucidate chronic morphological adaptations. Additionally, the predominance of male participants (83.7%) and methodological inconsistency in strength assessments (e.g., isokinetic vs. 1RM testing) limit the generalizability of current findings. Athletes should prioritize performance-oriented absolute pressure targets, whereas clinical and rehabilitative settings should favor AOP-guided titration methods to mitigate cardiovascular risk. These should be complemented by wearable near-infrared spectroscopy to ensure tissue oxygen saturation (TSI) remains above 30%. Future research must address these gaps by developing standardized BFR protocols, conducting sex-specific investigations, and incorporating AI-integrated BFR devices capable of real-time hemodynamic modulation. By integrating mechanistic insight with practical application frameworks, HL-BFRT holds transformative potential to redefine paradigms in both performance optimization and rehabilitative medicine.

## Data Availability

The original contributions presented in the study are included in the article/Supplementary Material, further inquiries can be directed to the corresponding author.
