# Supplementary material for: Effects of blood flow restriction combined with high-load training on muscle strength and sports performance in athletes: a systematic review and meta-analysis
Source: Front Physiol. 2025 Jul 2;16:1603568. doi: 10.3389/fphys.2025.1603568 (PMC12263612; doi:10.3389/fphys.2025.1603568)
Supplement: Supplementary file 1 [file DataSheet1.docx]

**Supplementary material**

**Supplementary Table 1** Search terms for each database and results of the number of

documents

**Supplementary Table 2** Effect of moderator variables with 95% confidence intervals.

**Supplementary Table 3** The mean ± SD physical fitness parameters reported for the HL-BFRT and HL-RT in the included studies.

**Supplementary Table 1** Search terms for each database and results of the number of documents

| Database | Number | Search step |
| --- | --- | --- |
| All database | 887 |  |
| PubMed | 174 | (((("Resistance Training"[Mesh]) OR ("Exercise"[Mesh])) OR ((((strength training) OR (high intensity training)) OR (weight training)) OR (high load training))) AND (("Blood Flow Restriction Therapy"[Mesh]) OR ((((((blood flow occlusion) OR (occluded blood flow)) OR (blood flow restriction)) OR (restricted blood flow)) OR (vascular occlusion)) OR (vascular restriction)))) AND (("Athletes"[Mesh]) OR ((((Professional Athlete) OR (Elite Athlete)) OR (College Athlete)) OR (Player))) |
| Embase | 269 | **#1**  resistance AND training OR exercise OR (strength AND training) OR (high AND intensity AND training) OR (weight AND training) OR (high AND load AND training)  **#2**  blood AND flow AND restriction AND therapy OR (blood AND flow AND occlusion) OR (occluded AND blood AND flow) OR (blood AND flow AND restriction) OR (restricted AND blood AND flow) OR (vascular AND occlusion) OR (vascular AND restriction)  **#3**  athletes OR (professional AND athlete) OR (elite AND athlete) OR (college AND athlete) OR player  **#4**  #1 AND #2 AND #3 |
| Web of Science | 444 | **#1**  Athletes (Topic) or Professional Athlete (Topic) or Elite Athlete (Topic) or College Athlete (Topic) or player (Topic) and Preprint Citation Index (Exclude – Database)  **#2**  Blood Flow Restriction Therapy (Topic) or blood flow occlusion (Topic) or occluded blood flow (Topic) or blood flow restriction (Topic) or restricted blood flow (Topic) or vascular occlusion (Topic) or vascular restriction (Topic) and Preprint Citation Index (Exclude – Database)  **#3**  resistance training (Topic) or exercise (Topic) or strength training (Topic) or high intensity training (Topic) or weight training (Topic) or high load training (Topic) and Preprint Citation Index (Exclude – Database)  **#4**  #1 AND #2 AND #3 and Preprint Citation Index (Exclude – Database) |

**Supplementary Table 2** Effect of moderator variables with 95% confidence intervals.

| **Subgroup** | **N** | **SMD (95%CI)** | **Z** | **P** | **Weight (%)** | **Between Group** | | **Within Group** | |
| --- | --- | --- | --- | --- | --- | --- | --- | --- | --- |
|  |  |  |  |  |  | **I^2^ (%)** | **p** | **I^2^ (%)** | **p** |
| **Strength** | | | | | | | | | |
| **Isokinetic Strength** | **7/7/7/116** | **0.78 (0.14, 1.41)** | **2.41** | **0.02** | **400.4** | **0** | **0.81** | **57** | **0.03** |
| **IRM** | **9/9/9/172** | **0.69 (0.28, 1.09)** | **3.32** | **0.0009** | **59.6** |  |  | **35** | **0.14** |
| **≤ 6 weeks** | **8/8/8/152** | **0.80 (0.45, 1.15)** | **4.50** | **＜0.001** | **50.3** | **29.9** | **0.23** | **63** | **0.008** |
| **> 6 weeks** | **8/8/8/136** | **0.50 (0.15, 0.85)** | **2.79** | **0.005** | **49.7** |  |  | **0** | **0.48** |
| **< 3 times/week** | **5/5/5/100** | **0.33 (-0.07, 0.73)** | **1.63** | **0.10** | **35.7** | **72** | **0.06** | **0** | **0.62** |
| **≥3 times/week** | **11/11/11/188** | **0.92 (0.48, 1.38)** | **3.91** | **＜0.001** | **64.3** |  |  | **45** | **0.03** |
| **Individualized Pressure** | **5/5/5/92** | **0.62 (-0.15, 1.39)** | **1.57** | **0.12** | **30.7** | **0** | **0.76** | **64** | **0.02** |
| **Absolute Pressure** | **11/11/11/196** | **0.75 (0.38, 1.12)** | **3.98** | **＜0.001** | **69.3** |  |  | **33** | **0.14** |
| **Power** | | | | | | | | | |
| **≤ 6 weeks** | **3/3/3/60** | **0.62 (0.09, 1.15)** | **2.31** | **0.02** | **69.8** | **20.4** | **0.26** | **0** | **0.40** |
| **> 6 weeks** | **2/2/2/24** | **0.07 (-0.73, 0.87)** | **0.18** | **0.86** | **30.2** |  |  | **0** | **0.98** |
| **Individualized Pressure** | **2/2/2/40** | **0.39 (-0.24, 1.02)** | **1.21** | **0.22** | **49.1** | **0** | **0.77** | **0** | **0.77** |
| **Absolute Pressure** | **3/3/3/44** | **0.52 (-0.10, 1.13)** | **1.65** | **0.10** | **50.9** |  |  | **32** | **0.23** |
| **Speed** | | | | | | | | | |
| **Individualized Pressure** | **3/3/3/50** | **0.30 (-0.38, 0.99)** | **0.87** | **0.39** | **59.1** | **70.1** | **0.07** | **29** | **0..25** |
| **Absolute Pressure** | **2/2/2/44** | **1.38 (0.45, 2.30)** | **2.92** | **0.003** | **40.9** |  |  | **44** | **0.18** |
| **Endurance** | | | | | | | | | |
| **Individualized Pressure** | **2/2/2/24** | **0.44 (-0.37, 1.26)** | **1.07** | **0.29** | **45.7** | **10.2** | **0.29** | **0** | **0.79** |
| **Absolute Pressure** | **2/2/2/43** | **1.29 (-0.05, 2.64)** | **1.88** | **0.06** | **54.3** |  |  | **74** | **0.05** |

| **Supplementary Table 3** The mean ± SD physical fitness parameters reported for the HL-BFRT and HL-RT in the included studies. | | | | | | | | | |
| --- | --- | --- | --- | --- | --- | --- | --- | --- | --- |
| **References** | **Parameters** | **HL-BFRT** | | | | **HL-RT** | | | |
|  |  | **Pre** | **Post** | **n** | **Change** | **Pre** | **Post** | **n** | **Change** |
| **Wang et al., 2022** | **PKE-60°/s (Nm)**  **PKF-60°/s (Nm)**  **Squat 1RM (kg)**  **SJ (cm)**  **TFT（cm）** | **213.1 ± 11.5**  **133.9 ± 6.1**  **190.2 ± 46.3**  **45.45 ± 7.03**  **57.90 ± 8.34** | **250.8 ± 13.5**  **155.2 ± 7.1**  **244.6 ± 59.6**  **47.5 ± 7.20**  **60.0 ± 8.50** | **6**  **6**  **6**  **6**  **6** | **37.7 ± 7.2**  **21.3 ± 3.8**  **54.3 ± 30.8**  **2.05±10.06**  **2.1±11.9** | **208.8 ± 9.6**  **131.7 ± 6.1**  **196.7 ± 52.7**  **43.00 ± 3.60**  **59.18 ± 3.72** | **235.7 ± 7.5**  **148.7 ± 6.9**  **222.1 ± 59.5**  **44.5 ± 3.80**  **60.5 ± 3.90** | **6**  **6**  **6**  **6**  **6** | **26.9 ± 4.9**  **17.0 ± 3.8**  **34.0 ± 32.5**  **1.5±5.2**  **1.3±5.4** |
| **Amani-Shalamzari et al., 2019** | **PKE-60°/s(Nm)**  **PKF-60°/s (Nm)**  **FSP (s)** | **185.8 ± 22.0**  **83.2 ± 13.1**  **34.9 ± 2.5** | **242.0 ± 12.7**  **102.3 ± 9.6**  **30.4 ± 2.6** | **6**  **6**  **6** | **56.2 ± 10.4**  **19.1 ± 6.6**  **-4.5 ± 1.9** | **190.9 ± 20.6**  **81.4 ± 8.3**  **33.8 ± 3.1** | **218.5 ± 9.2**  **87.7 ± 5.3**  **31.8 ± 3.2** | **6**  **6**  **6** | **27.6 ± 9.2**  **6.3 ± 4.0**  **-2.0 ± 1.8** |
| **Amani-Shalamzari et al., 2020** | **VO_2max_ (ml/min/kg)**  **RP (km/h)** | **44.0 ± 5.7**  **15.8 ± 0.9** | **48.7 ± 5.3**  **16.4 ± 1.1** | **6**  **6** | **4.7 ± 3.17**  **0.6 ± 0.6** | **37.1 ± 5.2**  **14.9 ± 0.7** | **39.8 ± 6.6**  **15.3 ± 0.9** | **6**  **6** | **2.7 ± 3.43**  **0.4 ± 0.5** |
| **Godawa et al., 2012** | **Bench press 1RM (kg)**  **Squat 1RM (kg)**  **Dead lift(kg)**  **Powerlifting Big Three(kg)**  **BM (kg)** | **147.9 ± 37.6**  **231.3 ± 50.8**  **227.8 ± 32.18**  **605.7± 115.43**  **89.97 ± 22.52** | **150.6 ± 34.9**  **245.8 ± 51.3**  **237.36 ± 32.18**  **630.85±113.89**  **88.75 ± 0.0** | **8**  **8**  **8**  **8**  **8** | **2.7 ± 18.1**  **14.5 ± 25.5**  **9.53±16.09**  **25.07±57.33**  **1.21 ± 7.96** | **104.8 ± 43.5**  **153.8 ± 52.6**  **170.62±63.50**  **429.17±155.62**  **77.52 ± 17.79** | **107.0 ± 43.1**  **157.4 ± 55.3**  **179.64±59.65**  **443.19±155.62**  **75.85 ± 0.0** | **10**  **10**  **10**  **10**  **10** | **2.2 ± 19.4**  **3.6 ± 24.1**  **9.02±27.55**  **14.02±69.59**  **1.21 ± 5.62** |
| **Cook et al., 2014** | **Bench press 1RM (kg)**  **Squat 1RM (kg)**  **40 m (s)**  **CMJ power (W)** | **139.0 ± 7.8**  **171.5 ± 11.9**  **5.08 ± 0.18**  **5216 ± 1027** | **NR**  **NR**  **NR**  **NR** | **10**  **10**  **10**  **10** | **5.3 ± 2.2**  **7.8 ± 2.1**  **-0.03 ± 0.01**  **168 ± 105** | **141.0 ± 13.6**  **174.8 ± 13.6**  **5.11 ± 0.18**  **5551 ± 932** | **NR**  **NR**  **NR**  **NR** | **10**  **10**  **10**  **10** | **3.3 ± 3.6**  **4.3 ± 1.4**  **-0.01 ± 0.01**  **68 ± 50** |
| **Elgammal et al., 2020** | **Bench press 1RM (kg)**  **Squat 1RM (kg)**  **143.3 m (s)**  **VO_2max_ (ml/min/kg)** | **76.5 ± 6.0**  **127.5 ± 10.1**  **32.3 ± 0.7**  **38.3 ± 2.2** | **87.3 ± 6.9**  **150.1 ± 10.6**  **32.1 ± 0.8**  **46.2 ± 3.1** | **12**  **12**  **12**  **12** | **10.8 ± 2.6**  **22.7 ± 8.2**  **-0.3 ± 0.1**  **7.9 ± 1.09** | **74.8 ± 5.5**  **126.0 ± 7.3**  **32.5 ± 0.6**  **38.1 ± 1.5** | **82.1 ± 6.6**  **140.4 ± 7.5**  **32.3 ± 0.6**  **43.9 ± 2.9** | **12**  **12**  **12**  **12** | **7.3 ± 2.5**  **14.4 ± 6.0**  **-0.2 ± 0.1**  **5.8 ± 0.94** |
| **Giovanna et al., 2022** | **VO_2max_ (ml/min/kg)** | **51.9 ± 4.6** | **54.8 ± 7.7** | **10** | **2.9 ± 2.83** | **54.0 ± 6.9** | **54.9 ± 7.3** | **9** | **0.9 ± 3.34** |
| **Lambert et al., 2023** | **Isometric Strength IR 90°(kg)**  **Shoulder Region Lean Mass(g)**  **Lean Mass(kg)** | **NR**  **NR**  **NR** | **NR**  **NR**  **NR** | **15**  **15**  **15** | **2.4 ± 2.3**  **227 ± 60**  **1.33±0.31** | **NR**  **NR**  **NR** | **NR**  **NR**  **NR** | **13**  **13**  **13** | **0.9±0.59**  **75±37**  **1.29±0.51** |
| **Pişkin et al., 2024** | **Dominant Leg Peak Torque (W)**  **Non-dominant Leg Peak Torque (W)**  **Dominant Leg Average Power (W)**  **Non-dominant Leg Average Power (W)** | **165.8 *±* 38.2**  **146.4 *±* 38.6**  **147.5 *±* 30.7**  **137.2 *±* 29.0** | **241.1 ± 52.6**  **228.4 *±* 46.3**  **229.9 *±* 51.9**  **217.3 *±* 49.9** | **10**  **10**  **10**  **10** | **75.3±14.3**  **82.0 ± 7.7**  **82.4 ± 21.2**  **80.1 ± 20.9** | **144.6 ± 48.9**  **135.1 *±* 51.8**  **135.5 *±* 42.3**  **127.7 *±* 46.4** | **220.9 ± 30.5**  **216.1 *±* 26.2**  **208.6 *±* 27.2**  **201.2 *±* 24.6** | **10**  **10**  **10**  **10** | **76.3±18.0**  **81.0 ± 25.6**  **73.1 ± 15.1**  **73.5 ± 21.8** |
| **Sander et al., 2024** | **Average Speed(m/s)**  **50m(s)** | **1.47 ± 0.19**  **34.8 ± 3.7** | **1.52 ± 0.16**  **33.9 ± 3.5** | **10**  **10** | **0.05±0.25**  **-0.9±1.61** | **1.40 ± 0.10**  **35.9 ± 3.4** | **1.44 ± 0.08**  **35.0 ± 3.3** | **9**  **9** | **0.04±0.13**  **-0.9±1.58** |

N, data denote the number of studies providing data for analysis, the number of HL-BFRT groups, the number of HL-RT groups and the total number of athletes included in the analysis, respectively; 1RM, 1-repetition maximum; CMJ, counter movement jump; VO2max, maximal oxygen consumption; PKF, peak knee flexion; PKE, peak knee extension; TFT, three footed takeoff; SJ, squat jump; RP, running performance; FSP, futsal special performance.
